# Dissecting the Methylomes of *EGFR*-Amplified Glioblastoma Reveals Altered DNA Replication and Packaging, and Chromatin and Gene Silencing Pathways

**DOI:** 10.3390/cancers15133525

**Published:** 2023-07-07

**Authors:** Theo F. J. Kraus, Celina K. Langwieder, Dorothee Hölzl, Georg Hutarew, Hans U. Schlicker, Beate Alinger-Scharinger, Christoph Schwartz, Karl Sotlar

**Affiliations:** 1 Institute of Pathology, University Hospital Salzburg, Paracelsus Medical University, Müllner Hauptstr. 48, A-5020 Salzburg, Austria; 2 Department of Neurosurgery, University Hospital Salzburg, Paracelsus Medical University, Ignaz-Harrer-Str. 79, A-5020 Salzburg, Austria

**Keywords:** glioblastoma, epigenetic profiling, methylome, *EGFR*, precision medicine

## Abstract

**Simple Summary:**

Glioblastoma is the most malignant brain tumor. To date, there is no curative therapy available. Since *EGFR* is an interesting candidate in precision medicine, we performed an integrated molecular analysis in glioblastoma with and without *EGFR* amplification. We found that there are significant molecular differences in glioblastoma, depending on the *EGFR* amplification state. Analysis of top differences revealed DNA replication and packaging, and chromatin and gene silencing pathways. Targeting these altered pathways may open novel targets in precision medicine.

**Abstract:**

Glioblastoma IDH wildtype is the most frequent brain tumor in adults. It shows a highly malignant behavior and devastating outcomes. To date, there is still no targeted therapy available; thus, patients’ median survival is limited to 12–15 months. Epithelial growth factor receptor (*EGFR*) is an interesting targetable candidate in advanced precision medicine for brain tumor patients. In this study, we performed integrated epigenome-wide DNA-methylation profiling of 866,895 methylation specific sites in 50 glioblastoma IDH wildtype samples, comparing *EGFR* amplified and non-amplified glioblastomas. We found 9849 significantly differentially methylated CpGs (DMCGs) with Δβ ≥ 0.1 and *p*-value < 0.05 in *EGFR* amplified, compared to *EGFR* non-amplified glioblastomas. Of these DMCGs, 2380 were annotated with tiling (2090), promoter (117), gene (69) and CpG islands (104); 7460 are located at other loci. Interestingly, the list of differentially methylated genes allocated eleven functionally relevant RNAs: five miRNAs (miR1180, miR1255B1, miR126, miR128-2, miR3125), two long non-coding RNAs (LINC00474, LINC01091), and four antisense RNAs (EPN2-AS1, MNX1-AS2, NKX2-2-AS1, WWTR1-AS1). Gene ontology (GO) analysis showed enrichment of “DNA replication-dependent nucleosome assembly”, “chromatin silencing at rDNA”, “regulation of gene silencing by miRNA”, “DNA packaging”, “posttranscriptional gene silencing”, “gene silencing by RNA”, “negative regulation of gene expression, epigenetic”, “regulation of gene silencing”, “protein-DNA complex subunit organization”, and “DNA replication-independent nucleosome organization” pathways being hypomethylated in *EGFR* amplified glioblastomas. In summary, dissecting the methylomes of *EGFR* amplified and non-amplified glioblastomas revealed altered DNA replication, DNA packaging, chromatin silencing and gene silencing pathways, opening potential novel targets for future precision medicine.

## 1. Introduction

The 2021 World Health Organization (WHO) classification for CNS tumors combines morphology and molecular pathology [1]. Thereby, the entities of gliomas, neuronal and glioneuronal tumors are assigned to six main groups, with focus on the underlying tumor biology: diffuse gliomas adult type; low-grade diffuse gliomas pediatric type; high-grade diffuse gliomas, pediatric type; circumscribed astrocytic gliomas; neuronal/glioneuronal tumors; and ependymal tumors [1]. Diffuse gliomas, adult type, comprise the groups astrocytomas IDH mutant, oligodendroglioma IDH mutant 1p/19q co-deleted, and glioblastoma IDH wildtype [1]. Low-grade diffuse gliomas, pediatric type, comprise the groups diffuse astrocytoma with MYB or MYBL1 alterations, angiocentric glioma, polymorphous low-grade neuroepithelial tumor of the young (PLNTY), and diffuse low-grade glioma with MAPK altered pathway [1]. High-grade diffuse pediatric type gliomas comprise the groups diffuse midline glioma with H3 K27 alteration, diffuse hemispheric glioma with H3 G34 mutations, pediatric type diffuse high-grade glioma with H3- and IDH-wildtype, and infant-type hemispheric glioma [1]. Circumscribed astrocytic gliomas comprise the groups pilocytic astrocytoma, high-grade astrocytoma with piloid features, pleomorphic xanthoastrocytoma (PXA), subependymal giant cell astrocytoma (SEGA), chordoid glioma, and astroblastoma with MN1 alteration [1]. Neuronal/glioneuronal tumors comprise the groups ganglioglioma, gangliocytoma, desmoplastic infantile ganglioglioma and astrocytoma, dysembryoplastic neuroepithelial tumor (DNT), diffuse glioneuronal tumor with oligodendroglioma-like features and nuclear clusters (DGONC), papillary glioneuronal tumor (PGNT), rosette-forming glioneuronal tumor (RGNT), myxoid glioneuronal tumor, diffuse leptomeningeal glioneuronal tumor (DLGNT), multinodular and vacuolating neuronal tumor (MVNT), Lhermitte—Duclos disease/dysplastic cerebellar gangliocytoma, central neurocytoma, extraventricular neurocytoma, and cerebellar liponeurocytoma [1]. Ependymal tumors comprise the groups supratentorial ependymoma NOS, supratentorial ependymoma with ZFTA fusion, supratentorial ependymoma with YAP1 fusion, posterior fossa ependymoma NOS, posterior fossa group A ependymoma (PFA), fosterior fossa group B ependymoma (PFB), spinal ependymoma NOS, spinal ependymoma with MYCN amplification, myxopapillary ependymoma, and subependymoma [1]. This novel approach emphasizes the inclusion of the biological background of the tumors in brain tumor classification, including the molecular hallmarks of gliomagenesis and tumor progression.

Focusing on gliomas of adults being allocated to astrocytomas, oligodendrogliomas, and glioblastomas, the main molecular hallmarks are as follows [1]: astrocytomas harbor isocitrate dehydrogenase (IDH) 1/2 mutations; and oligodendrogliomas harbor loss of 1p and 19q (1p/19q) combined with IDH1/2 mutations. Diffuse gliomas with *IDH1/2*, *H3F3A* (Histone H3 Family 3A) and *HIST1H3B/C* wildtype status are classified as glioblastoma [1]. Thereby, the degree of anaplasia is represented by the addition of a CNS WHO grade, depending on the degree of anaplasia, i.e., the presence of mitoses, microvascular proliferation, and necrosis: astrocytomas IDH mutated are graded as CNS WHO grade 2 to 4, oligodendrogliomas IDH mutated 1p/19q co-deleted are graded as CNS WHO grade 2 to 3, and glioblastomas IDH wildtype are graded as CNS WHO grade 4 [1].

Of all tumors of the CNS, glioblastomas IDH wildtype are the most malignant brain tumors in adults [1]. With 3–4 reported cases per 100,000 population in Western world countries, glioblastomas are also the most frequently diagnosed primary brain tumor [1]. The discovery of the methylation of the O6-methylguanin-DNA-methyltransferase (MGMT) promoter in 2008 opened up new possibilities in glioblastoma therapy [2,3,4]: hypermethylation of the MGMT promoter is associated with significantly longer survival in patients that receive an adjuvant radio-chemotherapy with temozolomide, according to the EORTC/NCIC protocol [5]. However, besides the addition of adjuvant tumor treating field therapy, to date, no further significant progresses in glioblastoma therapy has been made [1].

A promising novel molecular target for individualized patient care is the amplification of epidermal growth factor receptor (*EGFR*) [6]. *EGFR* is a receptor tyrosine kinase [7] contributing to cell proliferation and differentiation [7,8], bound to the epidermal growth factor (EGF) and other growth factors [9,10]. Genomic alterations of *EGFR* result in signaling independent of physiological ligands propagating pro-oncogenic processes [10]. In case of *EGFR* amplifications, there is a direct impact on protein levels with overexpressed EGFR protein [11]. Thus, detection of *EGFR* amplification, and subsequently altered pathways offers novel targets in molecular glioblastoma therapy.

Here, we performed an integrated analysis of 50 glioblastomas IDH wildtype with or without *EGFR* gene amplification, and performed epigenome-wide methylation analysis to reveal distinct differences in the glioblastoma methylome and subsequently affected pathways.

## 2. Materials and Methods

### 2.1. Tissue Collection

We analyzed 50 anonymized glioblastoma IDH wildtype CNS WHO grade 4 (Supplementary Table S1). Integrated diagnosis and re-classification were performed according to the 2021 CNS WHO classification including histology, immunohistochemistry, and molecular genetics [1]. We used formalin-fixed and paraffin-embedded (FFPE) tissue samples that underwent routine histological analysis and, subsequently, were stored in the archive of the University Institute of Pathology, University Hospital Salzburg, Paracelsus Medical University. Prior to study inclusion, samples were anonymized (non-identifiable samples).

### 2.2. Molecular Genetic Analysis

Molecular genetic analyses were performed as previously described [12,13,14,15]. Molecular genetic testing was performed according to the 2021 CNS WHO classification [1]. Thereby, essential diagnostic criteria for glioblastoma IDH wildtype are an IDH wildtype H3 wildtype diffuse astrocytic glioma, with one or more of the following criteria: microvascular proliferation, necrosis, TERT promoter mutation, EGFR gene amplification, and/or +7/−10 chromosome copy-number alterations. As further desirable criteria, there is a DNA methylation profile of glioblastoma IDH wildtype. Thus, in the cases with *TERT* promoter wild type status, or without information on *TERT* promoter mutations status (NA), diagnosis of glioblastoma IDH wildtype was assessed by applying the essential criteria of IDH wildtype H3 wildtype diffuse astrocytic glioma with the morphologic features of microvascular proliferation and necrosis, and the desirable criteria of a DNA methylation profile of glioblastoma IDH wildtype were applied. Due to this, we did not perform further molecular analyses on *TERT* promoter mutations in cases with unknown *TERT* status (4 cases), and we did not perform +7/−10 chromosome copy-number alteration analyses in cases with TERT wild type status (3 cases) (Supplementary Table S1).

Raw data files (idat-files) of Infinium Methylation Bead Chips were analyzed using the current brain tumor classifier of the molecularneuropathology.org bioinformatics pipeline [16]. Amplifications of copy-number variation (CNV) plots were interpreted as intensities of more than 0.6 on the log2-scale after baseline correction [14,17].

### 2.3. Computational Data Analysis

We used the Genome Studio (Illumina, San Diego, CA, USA) and RnBeads [18] for methylation analysis, as previously described with identical analysis parameters [19]. Analogous to other epigenome-wide association studies (EWAS), we used nominal *p*-values [20,21]. Further data visualization was performed applying GraphPad Prism 9 software suite and Morpheus.

## 3. Results

### 3.1. A Subfraction of Glioblastomas IDH Wildtype CNS WHO Grade 4 Shows EGFR Gene Amplification

**Figure 1:**
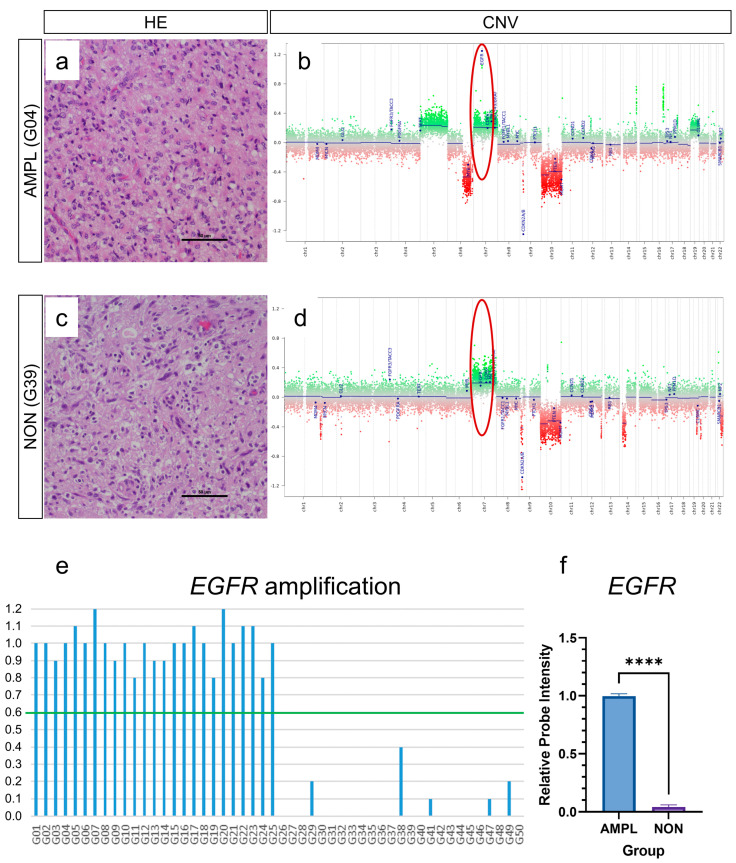
Analysis of *EGFR* amplification. Analysis of *EGFR* amplification in glioblastoma IDH wildtype CNS WHO grade 4 was performed by evaluating relative probe intensities of copy number profiles generated during methylation analysis. Fifty glioblastoma samples were included, with twenty-five samples showing *EGFR* amplification (i.e., relative probe intensities of more than 0.6, indicated by red circle) (**a**,**b**), and twenty-five showing no *EGFR* amplification (i.e., relative probe intensities of less than 0.6, indicated by red circle) (**c**,**d**). Of all glioblastomas, (**e**) mean *EGFR* probe intensity level of amplified glioblastomas was 0.99, and of non-amplified was 0.04 (**f**). (**a**,**c**): scale bar: 50 µm; **** *p* < 0.0001.

We performed an integrated analysis on glioblastomas IDH wildtype CNS WHO grade 4 that were processed in routine diagnostic workup. Analyses of copy number variation (CNV) plots that were checked routinely during sample workup (e.g., 1p and 19q losses) found that a subfraction of glioblastomas showed EGFR amplification. For this study, we subsequently selected 50 glioblastomas with known EGFR amplification status: 25 with amplified EGFR gene region (Figure 1a,b), and 25 without amplified gene region (Figure 1c,d). Thereby, intensities of more than 0.6 on the log2-scale after baseline correction were interpreted as *EGFR* amplifications, the correction of the baseline of chromosome 7 enabled distinguishing between chromosome 7 polysomy and *EGFR* amplification, as described by Stichel et al. [17].

Of all 50 glioblastomas (Figure 1e), the mean EGFR probe intensity levels (reflecting EGFR gene amplification) of amplified glioblastomas was 0.99, while the mean EGFR probe intensity levels of non-amplified glioblastomas was 0.04 (Figure 1f). These samples were subsequently processed on epigenome-wide DNA-methylome analysis. Analysis on molecular glioblastoma subtypes (mesenchymal, RTK I, RTK II) showed that all three molecular subtypes were evenly distributed in the analyzed cohort (Supplementary Figure S1a); however, there was a predominance of RTK II subtype in EGFR amplified glioblastomas (60%, Supplementary Figure S1b) and a predominance of RTK I subtype in EGFR non-amplified glioblastomas (48%, Supplementary Figure S1c). Further CNV alterations were not analyzed in the current study.

### 3.2. Differential Methylation Analysis of EGFR Amplified Glioblastomas IDH Wildtype CNS WHO Grade 4 Shows Distinct Epigenomic Alterations

**Figure 2:**
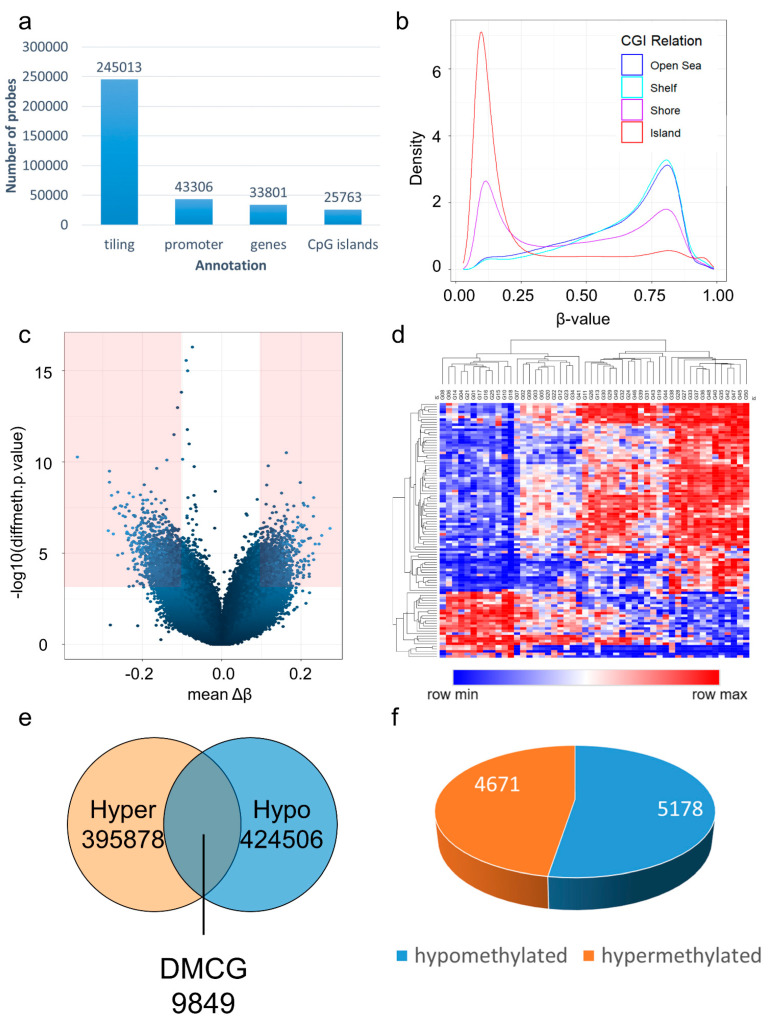
Exploratory methylation analysis. After probe removal and filtering, exploratory methylation analysis was performed on 820,384 probes that remained: 245,013 were annotated with tiling regions, 43,306 were annotated with promoters, 33,801 were annotated with genes, and 25,763 were annotated with CpG islands (**a**). Analysis of beta value distributions and probe categories showed that CpG islands represent high densities of unmethylated beta values, while shelf and open sea regions showed higher densities of methylated values with an intermediate distribution of shores (**b**). Volcano plot showed distribution of differential methylation values (Δβ) and significance level (−log10); thereby, probes with Δβ ≥ 0.1 and *p*-value < 0.05 are indicated in red color (**c**). Hierarchical clustering of top 100 DMCGs showed distinct clusters using Manhattan distance (**d**). Each pairwise comparison of probes resulted in 9849 significantly differentially methylated probes (Δβ ≥ 0.1, *p*-value < 0.05) (**e**), with 5178 probes being hypermethylated and 4671 probes being hypomethylated (**f**).

By using the Illumina Infinium EPIC bead chip, we interrogated 866,895 probes per sample. During data preprocessing, we removed 17,371 probes overlapping with SNPs, 7508 probes during greedy cut filtering, 2937 context specific probes, and 18,695 probes located on sex-chromosomes; of the remaining 820,384 probes, 245,013 were annotated with tiling regions, 43,306 were annotated with promoters, 33,801 were annotated with genes, and 25,763 were annotated with CpG islands (Figure 2a). Beta value distributions showed high densities of unmethylated methylation values in CpG islands (Figure 2b). Generation of a volcano plot showed the distribution of differential methylation values (Δβ values) and significance level (−log10); probes with Δβ values of ≥0.1 and *p*-value < 0.05 are indicated in red color (Figure 2c). Subsequent hierarchical clustering of the top 100 differentially methylated CpGs (DMCG) showed distinct clusters in the generated heat map using Manhattan distance (Figure 2d). Each pairwise comparison of probes resulted in 9849 significantly differentially methylated probes, with Δβ values of≥ 0.1 and *p*-value < 0.05 (Figure 2e); thereby, 5178 probes were hypermethylated and 4671 probes were hypomethylated (Figure 2f).

### 3.3. Enrichment Analysis of DMCGs Reveals Distinct Altered Pathways Correlating with EGFR Amplification Status

**Figure 3:**
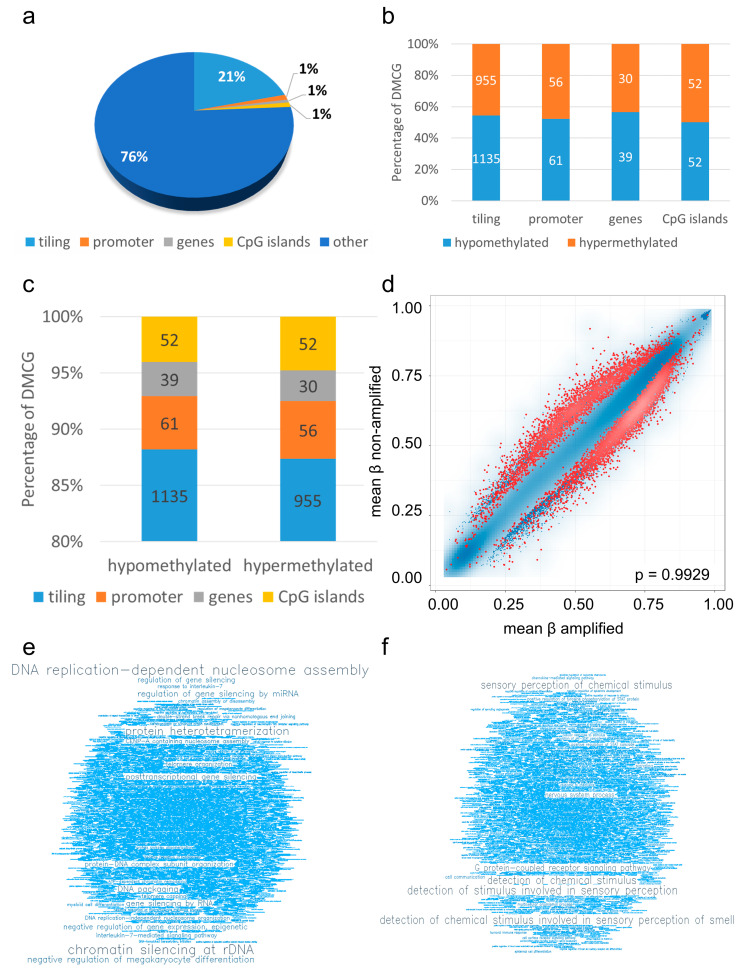
Differential methylation analysis. Differential methylation analysis revealed 2380 DMCGs that are annotated with tiling regions (2090, 21%), promoter regions (117, 1%), gene regions (69, 1%), and CpG island regions (104, 1%) (**a**), with 1287 being hypo- and 1093 being hypermethylated (**b**). Analysis of DMCG and genomic position showed distinct fractions of hypomethylated and hypermethylated DMCGs (**c**). Scatter plot of GO enrichment analysis indicated ranked regions with FDR adjusted *p*-values < 0.05 in red color (**d**), FDR adjusted *p*-values ≥ 0.05 in blue dots, FDR adjusted *p*-values < 0.05 in red dots). Results of GO analysis of top 1000 best ranking genes are demonstrated by word clouds of top terms being enriched in hypomethylated (**e**), and hypermethylated pathways in *EGFR* amplified glioblastomas (**f**).

Next, we performed gene enrichment analysis of identified DMCGs. Of the 9849 significantly DMCG, 2380 are annotated with 2090 tiling regions (21%), 117 promoter regions (1%), 69 gene regions (1%), and 104 CpG island regions (1%) (Figure 3a), and 7460 are located at other loci.

Interestingly, of the 2380 DMCGs, 1287 are hypo- and 1093 hypermethylated (Figure 3b). An analysis of DMCG and genomic position showed that, of 1287 significantly hypomethylated CpGs, 1135 are located in tiling regions (88%), 61 in promoter regions (5%), 39 in gene regions (3%) and 52 in CpG island regions (4%), and of all 1093 significantly hypermethylated CpGs, 955 are located in tiling regions (87%), 56 in promoter regions (5%), 30 in gene regions (3%) and 52 in CpG island regions (5%) (Figure 3c).

A closer look at the list of significant differentially methylated genes reveals that the top hypo- and hypermethylated genes are functionally important RNAs (Supplementary Table S2). By calculating the Δβ values (mean.mean.diff = mean.mean.AMPL − mean.mean.NON), we found that at the top position of all hypomethylated genes (top position of all differentially methylated genes), micro-RNA miR128-2 is significantly hypomethylated in EGFR amplified glioblastomas (Δβ = −0.17, *p*-value = 0.0000033); at the top positions of all hypermethylated genes (top 2 positions of all differentially methylated genes), the antisense RNA NKX2-2-AS1 is significantly hypermethylated in EGFR amplified glioblastomas (Δβ = 0.17, *p*-value = 0.0023), being hypomethylated in EGFR amplified glioblastomas (Supplementary Table S2). Interestingly, the list of differentially methylated genes allocated 11 functionally relevant RNAs: five miRNAs (miR1180, miR1255B1, miR126, miR128-2, miR3125), two long non-coding RNAs (LINC00474, LINC01091) and four antisense RNAs (EPN2-AS1, MNX1-AS2, NKX2-2-AS1, WWTR1-AS1) (Supplementary Table S2).

Performing gene ontology (GO) enrichment analysis, ranked regions with FDR (false discovery rate), adjusted *p*-values < 0.05 were identified (Figure 3d). GO term enrichments using the best 1000 ranking genes in the RnBeads pipeline highlighted pathways being associated with “DNA replication-dependent nucleosome assembly”, “chromatin silencing at rDNA”, “regulation of gene silencing by miRNA”, “DNA packaging”, “posttranscriptional gene silencing”, “gene silencing by RNA”, “negative regulation of gene expression, epigenetic”, “regulation of gene silencing”, “protein-DNA complex subunit organization” and “DNA replication-independent nucleosome organization” being hypomethylated (Figure 3e, Supplementary Table S3a), and “cell communication” and “signaling” being hypermethylated in EGFR amplified glioblastomas (Figure 3f, Supplementary Table S3b).

## 4. Discussion

Targeting *EGFR* overexpression may be a promising novel approach in glioblastoma therapy, since small molecule tyrosine kinase inhibitors (TKIs) and monoclonal antibodies have already been approved in other tumor entities [6].

We found that there are distinct, significantly differentially methylated regions in glioblastomas correlating with *EGFR* amplification status. We found 9849 significant DMCGs with Δβ values of ≥0.1 and *p*-value < 0.05 (Figure 2e). Of these, 5178 were hypermethylated and 4671 were hypomethylated in *EGFR* amplified glioblastomas (Figure 2f). Annotating these DMCGs to genomic positions, we found that they are annotated with 2090 tiling regions (21%), 117 promoter regions (1%), 69 gene regions (1%), and 104 CpG island regions (1%) (Figure 3a), with approximately even amounts of hypo- and hypermethylated CpGs in tiling, promoter, gene, and CpG island regions (Figure 3b).

Interestingly, the list of top significant differentially methylated genes reveals that the top hypo- and hypermethylated genes are both functionally important RNAs (Supplementary Table S2). The top position of hypermethylated genes in *EGFR* non-amplified glioblastomas (i.e., hypomethylated in *EGFR* amplified glioblastomas) is micro-RNA miR128-2, and the top position of hypomethylated genes in *EGFR* non-amplified glioblastomas (i.e., hypermethylated in *EGFR* amplified glioblastomas) is antisense RNA NKX2-2-AS1 (Supplementary Table S2).

According to Budi et al., miR128 is involved in cancer development, progression and therapy response [22]. Thereby, miR128 is associated with many different cellular processes, such as cell proliferation and self-renewal, chemotherapeutic resistance, cell growth and differentiation, angiogenesis and senescence, and apoptosis [22]. Decreased expression of miR128 has been described in a broad range of cancers, such as breast cancer, lung cancer, thyroid cancer, laryngeal cancer, head and neck cancer, melanoma, osteosarcoma, leukemia, and glioma [22]. Shi et al. found that overexpression of miR128 inhibits tumor growth and neovascularization through the miR128/p70S6K1 pathway [23]. Ye et al. found that miR128 promotes apoptosis, and that downregulation of miR128 expression reduced glioma cell death [24].

*NKX2.2* is also well known in the context of other tumor types, such as colorectal cancer, lung cancer, neuroendocrine tumors, osteosarcomas, Ewing sarcomas and brain tumors [25].

Cao et al. found that NKX2-2-AS1 was highly expressed in Ewing sarcomas, functioning as a core co-regulator of the driver mutation fusion gene, and is recognized as a specific biomarker for Ewing sarcomas [26]. Muraguchi et al. described that *NKX2-2* suppresses the self-renewal of glioma-initiating cells [27]. In glioma models, downregulation of *NKX2.2* was correlated with increased tumor malignancy and accelerated tumor growth and progression [27].

Focusing on further differentially methylated genes, it is of high interest that there are, in sum, eleven functionally relevant RNAs among top matches: five miRNAs (miR1180, miR1255B1, miR126, miR128-2, miR3125), two long non-coding RNAs (LINC00474, LINC01091), and four antisense RNAs (EPN2-AS1, MNX1-AS2, NKX2-2-AS1, WWTR1-AS1) (Supplementary Table S2). GO enrichment analysis showed enrichment of pathways being associated with “DNA replication-dependent nucleosome assembly”, “chromatin silencing at rDNA”, “regulation of gene silencing by miRNA”, “DNA packaging”, “posttranscriptional gene silencing”, “gene silencing by RNA”, “negative regulation of gene expression, epigenetic”, “regulation of gene silencing”, “protein-DNA complex subunit organization”, and “DNA replication-independent nucleosome organization” being hypomethylated (Figure 3e, Supplementary Table S3a), and “cell communication” and “signaling” being hypermethylated in *EGFR* amplified glioblastomas (Figure 3f, Supplementary Table S3b).

As a limitation of this study, the results were not validated in an independent cohort or using published data, such as TCGA data. This should be added in additional analyses, to increase the validity of results. Furthermore, correlation of methylation data with gene expression should be added in further studies to evaluate the biological relevance of methylation differences. Furthermore, in the current study, we used nominal *p*-values for analysis and did used *p*-values with correction for multiple testing; this is in accordance with other epigenome-wide association studies (EWAS) [20,21].

In summary, dissecting the methylomes of *EGFR* amplified and non-amplified glioblastomas revealed altered DNA replication, DNA packaging, chromatin silencing and gene silencing pathways, opening potential novel targets and targetable pathways for future precision medicine.

## 5. Conclusions

Despite intensive research, there still is no curative therapy for glioblastoma patients available. Since *EGFR* amplification is a promising novel candidate in precision medicine, we performed an epigenome-wide DNA-methylation analysis of *EGFR* amplified and non-amplified glioblastomas. We identified distinct DMCGs, depending on *EGFR* amplification status. Interestingly, the top DMCGs showed an overrepresentation of functionally relevant RNAs, and GO analysis showed enrichment of DNA replication, DNA packaging, chromatin silencing, and gene silencing pathways being hypomethylated in *EGFR* amplified glioblastomas.

## Data Availability

Data is available from the corresponding author on request.

## Supplementary Materials

The following supporting information can be downloaded at: https://www.mdpi.com/article/10.3390/cancers15133525/s1, Figure S1: Analysis on molecular glioblastoma subtypes; Table S1: Patient details; Table S2: Top significant differentially methylated genes; Table S3: GO enrichment analysis of top hypomethylated and hypermethylated pathways in *EGFR* amplified glioblastomas.
