# Supplementary material for: Dissecting the Methylomes of EGFR-Amplified Glioblastoma Reveals Altered DNA Replication and Packaging, and Chromatin and Gene Silencing Pathways
Source: Cancers (Basel). 2023 Jul 7;15(13):3525. doi: 10.3390/cancers15133525 (PMC10341082; doi:10.3390/cancers15133525)
Supplement: Supplementary file 1 [file cancers-15-03525-s001.zip › Table_S1.pdf]

Supplementary Table S1: Patient details. Indicated are details on analyzed patient samples, including age, sex, tumor type, molecular hallmarks and EGFR amplification level.

Sample-ID	Age [y]	Sex	2021 CNS WHO	Grade	Subgroup v11b4	IDH1	IDH2	TERT	MGMT	EGFR Relative Probe Intensity	EGFR-Status
G01	47	m	GBM IDH wt	4	RTK II	wt	wt	C250T	unmethylated	1.0	AMPL
G02	66	m	GBM IDH wt	4	Mesenchymal	wt	wt	C228T	methylated	1.0	AMPL
G03	74	m	GBM IDH wt	4	Mesenchymal	wt	wt	C250T	methylated	0.9	AMPL
G04	69	m	GBM IDH wt	4	RTK II	wt	wt	C228T	methylated	1.0	AMPL
G05	79	f	GBM IDH wt	4	Mesenchymal	wt	wt	C228T	unmethylated	1.1	AMPL
G06	77	f	GBM IDH wt	4	RTK II	wt	wt	C228T	unmethylated	1.0	AMPL
G07	49	m	GBM IDH wt	4	RTK II	wt	wt	C250T	methylated	1.2	AMPL
G08	63	w	GBM IDH wt	4	RTK II	wt	wt	C228T	methylated	1.0	AMPL
G09	65	m	GBM IDH wt	4	Mesenchymal	wt	wt	C228T	methylated	0.9	AMPL
G10	65	m	GBM IDH wt	4	RTK II	wt	wt	C228T	unmethylated	1.0	AMPL
G11	55	m	GBM IDH wt	4	RTK I	wt	wt	C228T	methylated	0.8	AMPL
G12	80	w	GBM IDH wt	4	RTK II	wt	wt	C228T	methylated	1.0	AMPL
G13	58	m	GBM IDH wt	4	Mesenchymal	wt	wt	C228T	methylated	0.9	AMPL
G14	64	m	GBM IDH wt	4	RTK II	wt	wt	C228T	methylated	1.0	AMPL
G15	86	m	GBM IDH wt	4	RTK II	wt	wt	C228T	methylated	1.0	AMPL
G16	41	m	GBM IDH wt	4	RTK II	wt	wt	C228T	unmethylated	1.0	AMPL
G17	77	m	GBM IDH wt	4	RTK II	wt	wt	C228T	methylated	1.1	AMPL
G18	48	m	GBM IDH wt	4	RTK II	wt	wt	C228T	methylated	1.0	AMPL
G19	52	w	GBM IDH wt	4	RTK I	wt	wt	C228T	methylated	0.8	AMPL
G20	55	m	GBM IDH wt	4	Mesenchymal	wt	wt	n.a.	methylated	1.2	AMPL
G21	71	m	GBM IDH wt	4	RTK II	wt	wt	C228T	methylated	1.0	AMPL
G22	54	m	GBM IDH wt	4	Mesenchymal	wt	wt	C228T	unmethylated	1.1	AMPL
G23	71	m	GBM IDH wt	4	RTK II	wt	wt	C250T	methylated	1.1	AMPL
G24	38	m	GBM IDH wt	4	Mesenchymal	wt	wt	n.a.	unmethylated	0.8	AMPL
G25	79	m	GBM IDH wt	4	RTK II	wt	wt	n.a.	methylated	1.0	AMPL
G26	70	f	GBM IDH wt	4	RTK II	wt	wt	C250T	methylated	0.0	NON
G27	62	m	GBM IDH wt	4	RTK II	wt	wt	C250T	methylated	0.0	NON
G28	77	m	GBM IDH wt	4	RTK I	wt	wt	C228T	unmethylated	0.0	NON
G29	43	f	GBM IDH wt	4	Mesenchymal	wt	wt	C250T	unmethylated	0.2	NON
G30	85	m	GBM IDH wt	4	Mesenchymal	wt	wt	C228T	methylated	0.0	NON

G31	63	f	GBM IDH wt	4	Mesenchymal	wt	wt	C250T	methylated	0.0	NON
G32	65	w	GBM IDH wt	4	Mesenchymal	wt	wt	n.a.	methylated	0.0	NON
G33	72	m	GBM IDH wt	4	RTK I	wt	wt	C228T	methylated	0.0	NON
G34	75	f	GBM IDH wt	4	RTK II	wt	wt	C250T	unmethylated	0.0	NON
G35	45	f	GBM IDH wt	4	RTK I	wt	wt	C228T	methylated	0.0	NON
G36	61	m	GBM IDH wt	4	RTK I	wt	wt	wt	unmethylated	0.0	NON
G37	51	m	GBM IDH wt	4	RTK I	wt	wt	C250T	methylated	0.0	NON
G38	63	f	GBM IDH wt	4	RTK I	wt	wt	C250T	methylated	0.4	NON
G39	65	f	GBM IDH wt	4	Mesenchymal	wt	wt	C228T	methylated	0.0	NON
G40	76	f	GBM IDH wt	4	RTK I	wt	wt	wt	methylated	0.0	NON
G41	72	f	GBM IDH wt	4	Mesenchymal	wt	wt	C250T	unmethylated	0.1	NON
G42	76	m	GBM IDH wt	4	RTK I	wt	wt	C228T	methylated	0.0	NON
G43	69	m	GBM IDH wt	4	RTK II	wt	wt	wt	methylated	0.0	NON
G44	62	m	GBM IDH wt	4	RTK I	wt	wt	C250T	unmethylated	0.0	NON
G45	69	m	GBM IDH wt	4	RTK I	wt	wt	C228T	unmethylated	0.0	NON
G46	68	w	GBM IDH wt	4	Mesenchymal	wt	wt	C250T	methylated	0.0	NON
G47	61	m	GBM IDH wt	4	RTK I	wt	wt	C228T	unmethylated	0.1	NON
G48	64	w	GBM IDH wt	4	Mesenchymal	wt	wt	C228T	methylated	0.0	NON
G49	76	m	GBM IDH wt	4	Mesenchymal	wt	wt	C228T	methylated	0.2	NON
G50	55	m	GBM IDH wt	4	RTK I	wt	wt	C228T	unmethylated	0.0	NON
