# Supplementary material for: Dissecting the Methylomes of EGFR-Amplified Glioblastoma Reveals Altered DNA Replication and Packaging, and Chromatin and Gene Silencing Pathways
Source: Cancers (Basel). 2023 Jul 7;15(13):3525. doi: 10.3390/cancers15133525 (PMC10341082; doi:10.3390/cancers15133525)
Supplement: Supplementary file 1 [file cancers-15-03525-s001.zip › Table_S2.pdf]

Supplementary Table S2. Top significant differentially methylated genes. Analysis of top significant differentially methylated genes showed micro-RNA miR128-2 as top 1 of all differentially methylated genes (and top 1 of all hypomethylated genes in EGFR amplified glioblastomas) with Δβ = -0.17 and p-value = 0.0000033 and antisense-RNA NKX2-2-AS1 as top 2 of all differentially methylated genes (and top 1 of all hypermethylated genes in EGFR amplified glioblastomas) with Δβ = 0.17 and p-value = 0.0023. It is of note that there are eleven functionally relevant RNAs (five miRNAs: miR1180, miR1255B1, miR126, miR128-2, miR3125; two long non-coding RNAs: LINC00474, LINC01091; four antisense RNAs: EPN2-AS1, MNX1-AS2, NKX2-2-AS1, WWTR1-AS1) among top hits. Methylation differences mean.mean.diff were determined by calculating mean.mean.AMPL - mean.mean.NON.

ID	Chromosome	Start	End	Symbol	Location	mean.mean.AMPL	mean.mean.NON	mean.mean.diff	mod.mean.mean.diff	comb.p.val	combinedRank
ENSG00000207625	chr3	35785968	35786051	MIR128-2	gene	0.322220972	0.492248572	-0.170027601	0.170027601	0.0000033	9
ENSG00000258197	chr20	21492085	21492947	NKX2-2-AS1	gene	0.422625332	0.255251283	0.16737405	0.16737405	0.002298411	165
ENSG00000232163	chr13	23947562	23948200	RPLP1P13	gene	0.514541929	0.680636707	-0.166094778	0.166094778	1.90383E-05	44
ENSG00000185899	chr7	143140546	143141502	TAS2R60	gene	0.75100281	0.590514665	0.160488145	0.160488145	3.30219E-05	82
ENSG00000224854	chr9	21967137	21967738	C9orf53	gene	0.372027307	0.528713639	-0.156686332	0.156686332	0.001853354	141
ENSG00000120738	chr5	137801179	137805004	EGR1	gene	0.385534577	0.54146755	-0.155932973	0.155932973	7.29307E-07	63
ENSG00000221540	chr17	19247819	19247887	MIR1180	gene	0.401727072	0.557241617	-0.155514545	0.155514545	0.000730704	91
ENSG00000229164	chr14	23016447	23021097	TRAC	gene	0.485156371	0.639491389	-0.154335018	0.154335018	0.019106428	819
ENSG00000255270	chr11	19402031	19406561	NAV2-IT1	gene	0.430165533	0.583579453	-0.15341392	0.15341392	0.000290236	48
ENSG00000252312	chr14	36700481	36702480	RN7SKP21	prom	0.592926542	0.745772051	-0.15284551	0.15284551	1.83862E-05	183
ENSG00000186960	chr14	29240410	29242409	C14orf23	prom	0.246484017	0.39716641	-0.150682394	0.150682394	8.97213E-05	50
ENSG00000264370	chr2	12875993	12877992	MIR3125	prom	0.574532903	0.721350724	-0.146817821	0.146817821	3.28119E-06	188
ENSG00000146859	chr7	134831324	134833323	TMEM140	prom	0.279612354	0.423797232	-0.144184878	0.144184878	0.000608082	145
ENSG00000207269	chr5	100067262	100069261	RN7SKP62	prom	0.362999258	0.505348162	-0.142348904	0.142348904	1.97458E-05	37
ENSG00000152467	chr19	58543900	58545899	ZSCAN1	prom	0.346455431	0.208809498	0.137645933	0.137645933	0.00383955	480
ENSG00000212409	chr9	113859194	113861193	RNY4P18	prom	0.701204568	0.563944151	0.137260417	0.137260417	4.10187E-05	231
ENSG00000188379	chr9	21384897	21386896	IFNA2	prom	0.473866383	0.336754375	0.137112008	0.137112008	9.34179E-06	29
ENSG00000202337	chr14	32671976	32673975	RNU6-8	prom	0.374319223	0.238511563	0.13580766	0.13580766	0.000272438	88
ENSG00000255287	chr11	92669107	92671106	SNRPGP16	prom	0.630782623	0.495180796	0.135601827	0.135601827	0.00039503	157
ENSG00000253279	chr8	29777529	29779528	FAM183CP	prom	0.812754818	0.677628921	0.135125897	0.135125897	1.33243E-06	377
ENSG00000235397	chr17	19199909	19209574	EPN2-AS1	gene	0.3457932	0.48012022	-0.13432702	0.13432702	0.000109001	25
ENSG00000179468	chr7	142723245	142724282	OR9A2	gene	0.710800645	0.577732763	0.133067883	0.133067883	0.000304987	139
ENSG00000253032	chr5	55419998	55421997	RNU6-299P	prom	0.558921016	0.690897479	-0.131976463	0.131976463	0.001723165	269
ENSG00000223118	chr2	124626648	124628647	RN7SKP102	prom	0.684477855	0.554662253	0.129815602	0.129815602	0.000116971	261
ENSG00000176293	chr19	58569107	58571106	ZNF135	prom	0.364420298	0.234807596	0.129612701	0.129612701	0.00014374	69
ENSG00000196832	chr14	20665495	20666605	OR11G2	gene	0.565664395	0.437418307	0.128246088	0.128246088	0.000939813	101
ENSG00000222300	chr2	159791998	159793997	RNU2-21P	prom	0.534004516	0.659875875	-0.125871359	0.125871359	0.000123403	155
ENSG00000132530	chr17	6657266	6659265	XAF1	prom	0.380460944	0.505781888	-0.125320944	0.125320944	4.15164E-05	52
ENSG00000255501	chr11	105008448	105010453	CARD18	gene	0.625243217	0.500583827	0.12465939	0.12465939	0.000398214	107
ENSG00000224854	chr9	21965637	21967636	C9orf53	prom	0.393401961	0.517680058	-0.124278097	0.124278097	0.001661056	265
ENSG00000235029	chr7	156799001	156799826	MNX1-AS2	gene	0.34813271	0.472171788	-0.124039078	0.124039078	0.006541228	353
ENSG00000123689	chr1	209848765	209849733	G0S2	gene	0.308852442	0.186918862	0.121933579	0.121933579	0.002447504	176
ENSG00000206923	chr9	113664117	113666116	RNU6-432P	prom	0.707323787	0.587968682	0.119355104	0.119355104	3.61317E-05	399
ENSG00000223037	chr4	165540384	165542383	RNU6-284P	prom	0.490782171	0.610044192	-0.119262021	0.119262021	1.6599E-05	223

ENSG00000266730	chr3	195647501	195649500	RN7SL773P	prom	0.393610458	0.512749694	-0.119139235	0.119139235	7.39771E-05	107
ENSG00000252613	chr21	18823499	18825498	RNU7-168P	prom	0.249497308	0.368229697	-0.118732389	0.118732389	0.002833082	381
ENSG00000168967	chr5	22140961	22142960	PMCHL1	prom	0.412144485	0.530463882	-0.118319397	0.118319397	0.000142555	89
ENSG00000251568	chr5	5375836	5376949	ALG3P1	gene	0.578093796	0.695861962	-0.117768166	0.117768166	0.002786153	215
ENSG00000251568	chr5	5376450	5378449	ALG3P1	prom	0.578093796	0.695861962	-0.117768166	0.117768166	0.002786153	407
ENSG00000241313	chr3	149373307	149375306	WWTR1-AS1	prom	0.371826289	0.489501448	-0.11767516	0.11767516	0.001448866	247
ENSG00000249464	chr4	124569922	124571921	LINC01091	prom	0.538950993	0.656059118	-0.117108125	0.117108125	4.85605E-05	263
ENSG00000138755	chr4	76922428	76928641	CXCL9	gene	0.634693466	0.51783409	0.116859376	0.116859376	0.000128379	144
ENSG00000213029	chr1	229440129	229441248	SPHAR	gene	0.525020334	0.641507763	-0.116487429	0.116487429	0.000146956	159
ENSG00000199940	chr7	71150233	71152232	RN7SKP75	prom	0.677087914	0.560680221	0.116407693	0.116407693	0.001106958	303
ENSG00000184617	chr20	45113100	45121279	ZNF840	gene	0.421583077	0.537841823	-0.116258746	0.116258746	0.002317249	167
ENSG00000162621	chr1	74977799	74979798	LRRC53	prom	0.397853897	0.513872931	-0.116019033	0.116019033	0.001203412	211
ENSG00000235289	chr2	70580142	70583047	BRD7P6	gene	0.725736678	0.610910219	0.114826458	0.114826458	0.000623857	276
ENSG00000214140	chr17	74522168	74524167	PRCD	prom	0.573845262	0.46006075	0.113784512	0.113784512	2.51663E-05	177
ENSG00000199161	chr9	139565054	139565138	MIR126	gene	0.486477611	0.373907952	0.112569659	0.112569659	0.036209346	1423
ENSG00000153071	chr5	39461903	39463902	DAB2	prom	0.492648808	0.604642746	-0.111993938	0.111993938	0.00112539	286
ENSG00000223770	chr7	81638493	81659271	MIR1255B1	gene	0.486452136	0.598423687	-0.111971551	0.111971551	0.000524429	133
ENSG00000189431	chr11	13030696	13032647	RASSF10	gene	0.415843355	0.305996532	0.109846823	0.109846823	0.015913128	700
ENSG00000124237	chr20	56724460	56726459	C20orf85	prom	0.563033427	0.454471829	0.108561598	0.108561598	0.000455291	146
ENSG00000214289	chr3	134074904	134076903	RPL39P5	prom	0.696415238	0.588421512	0.107993726	0.107993726	0.000116473	491
ENSG00000213538	chr11	9114410	9116409	KRT8P41	prom	0.66909915	0.561594	0.107505151	0.107505151	0.000528859	466
ENSG00000181894	chr19	58665978	58667977	ZNF329	prom	0.284294666	0.176909138	0.107385528	0.107385528	0.005425016	605
ENSG00000251934	chr11	3735606	3737605	RNU6-1143P	prom	0.746801526	0.640729716	0.10607181	0.10607181	0.003303656	732
ENSG00000235029	chr7	156797501	156799500	MNX1-AS2	prom	0.361706594	0.467343882	-0.105637288	0.105637288	0.033077948	2187
ENSG00000206199	chr3	149685673	149687672	ANKUB1	prom	0.500392529	0.395168605	0.105223924	0.105223924	0.002003663	299
ENSG00000248546	chr4	165118159	165118863	ANP32C	gene	0.660780157	0.765857749	-0.105077591	0.105077591	2.462E-05	345
ENSG00000223770	chr7	81636993	81638992	MIR1255B1	prom	0.486317341	0.591240401	-0.10492306	0.10492306	0.000242367	300
ENSG00000237111	chr14	106329626	106329675	IGHJ3P	gene	0.351192958	0.455537277	-0.104344319	0.104344319	0.006504893	352
ENSG00000122861	chr10	75667435	75669434	PLAU	prom	0.265889158	0.36998452	-0.104095361	0.104095361	0.000353182	103
ENSG00000188211	chr11	17371773	17373772	NCR3LG1	prom	0.616551565	0.5124758	0.104075765	0.104075765	0.005121566	578
ENSG00000160753	chr1	155289187	155291186	RUSC1	prom	0.333141837	0.437209947	-0.10406811	0.10406811	0.005417912	603
ENSG00000252887	chr16	89509726	89509832	RNU6-430P	gene	0.486771108	0.589747999	-0.10297689	0.10297689	0.000355891	189
ENSG00000228599	chr22	40502571	40504570	RPL7P52	prom	0.382876669	0.485804257	-0.102927588	0.102927588	0.000782145	165
ENSG00000214285	chr10	129347613	129350935	NPS	gene	0.506709083	0.609290763	-0.10258168	0.10258168	0.011948843	564
ENSG00000198131	chr19	58738460	58740459	ZNF544	prom	0.225285598	0.123187679	0.102097919	0.102097919	0.001631932	262
ENSG00000214533	chr2	65892329	65894328	KRT18P33	prom	0.715983988	0.614499746	0.101484242	0.101484242	0.000501343	735
ENSG00000139269	chr12	57844606	57846605	INHBE	prom	0.667535787	0.566463227	0.10107256	0.10107256	0.002137912	591
ENSG00000236460	chr7	31979374	31981373	SNX2P2	prom	0.567219589	0.466359181	0.100860408	0.100860408	0.005205645	584
ENSG00000204148	chr9	118686987	118688986	LINC00474	prom	0.661505094	0.560838896	0.100666198	0.100666198	0.002879812	577
ENSG00000229910	chr13	68876935	68878934	HNRNPA1P18	prom	0.539966523	0.439567887	0.100398636	0.100398636	0.004922156	560
