# Supplementary material for: Dissecting the Methylomes of EGFR-Amplified Glioblastoma Reveals Altered DNA Replication and Packaging, and Chromatin and Gene Silencing Pathways
Source: Cancers (Basel). 2023 Jul 7;15(13):3525. doi: 10.3390/cancers15133525 (PMC10341082; doi:10.3390/cancers15133525)
Supplement: Supplementary file 1 [file cancers-15-03525-s001.zip › Table_S3.pdf]

Supplementary Table S3. GO enrichment analysis of top hypomethylated and hypermethylated pathways in EGFR amplified glioblastomas. GO term enrichment analysis showed GO term enrichment of pathways being associated with “DNA replication-dependent nucleosome assembly”, “chromatin silencing at rDNA”, “regulation of gene silencing by miRNA”, “DNA packaging”, “posttranscriptional gene silencing”, “gene silencing by RNA”, “negative regulation of gene expression, epigenetic”, “regulation of gene silencing”, “protein-DNA complex subunit organization” and “DNA replication-independent nucleosome organization” being hypomethylated (a) and “cell communication” and “signaling” being hypermethylated (b) in EGFR amplified glioblastomas.

**(a) EGFR: amplified versus non-amplified: Hypomethylated Pathways**						
**GOMFID**	**Pvalue**	**OddsRatio**	**ExpCount**	**Count**	**Size**	**Term**
GO:0006335	0	195	0.5293	24	32	DNA replication-dependent nucleosome assembly
GO:0000183	0	112	0.6285	24	38	chromatin silencing at rDNA
GO:0051290	0	52	0.8932	24	54	protein heterotetramerization
GO:0045653	0	219	0.2977	14	18	negative regulation of megakaryocyte differentiation
GO:0060964	0	19	2	25	111	regulation of gene silencing by miRNA
GO:0006323	0	13	3	30	187	DNA packaging
GO:0016441	0	7	7	42	445	posttranscriptional gene silencing
GO:0031047	0	7	8	43	471	gene silencing by RNA
GO:0045814	0	15	2	24	128	negative regulation of gene expression, epigenetic
GO:0060968	0	13	3	25	152	regulation of gene silencing
GO:0071824	0	9	4	31	260	protein-DNA complex subunit organization
GO:0032200	0	11	3	24	170	telomere organization
GO:0034080	0	31	0.6947	14	42	CENP-A containing nucleosome assembly
GO:0038111	0	47	0.4631	12	28	interleukin-7-mediated signaling pathway
GO:0034724	0	23	0.8601	14	52	DNA replication-independent nucleosome organization
GO:0016233	0	21	0.9097	14	55	telomere capping
GO:0098760	0	30	0.612	12	37	response to interleukin-7
GO:0006303	0	14	1	15	82	double-strand break repair via nonhomologous end joining
GO:0030099	0	5	7	29	400	myeloid cell differentiation
GO:0006333	0	10	2	16	117	chromatin assembly or disassembly
GO:0043044	0	12	1	14	85	ATP-dependent chromatin remodeling
GO:0051259	0	4	9	31	557	protein complex oligomerization
GO:1903707	0	7	2	15	147	negative regulation of hemopoiesis
GO:0045652	0	13	0.9274	10	59	regulation of megakaryocyte differentiation
GO:0048534	0	3	15	39	895	hematopoietic or lymphoid organ development
GO:0010605	0	2	48	84	2882	negative regulation of macromolecule metabolic process
GO:0006352	0	5	4	17	236	DNA-templated transcription, initiation
GO:0045596	0	3	11	29	664	negative regulation of cell differentiation
GO:0045892	0	2	19	41	1141	negative regulation of transcription, DNA-templated
GO:0002376	0	2	46	76	2781	immune system process
GO:0051387	0	Inf	0.0496	3	3	negative regulation of neurotrophin TRK receptor signaling pathway
GO:0052470	0	Inf	0.0496	3	3	modulation by host of symbiont signal transduction pathway
GO:0052526	0	Inf	0.0496	3	3	positive regulation by organism of signal transduction in other organism involved in symbiotic interaction
GO:0075206	0	Inf	0.0496	3	3	positive regulation by host of symbiont cAMP-mediated signal transduction
GO:1902679	0	2	20	41	1187	negative regulation of RNA biosynthetic process
GO:0035195	0	4	5	17	323	gene silencing by miRNA
GO:0071345	0	2	17	36	1048	cellular response to cytokine stimulus

GO:0002227	0	18	0.3639	5	22	innate immune response in mucosa
GO:0048731	0	2	56	83	3556	system development
GO:0044501	0	90	0.0827	3	5	modulation of signal transduction in other organism
GO:0051343	0	90	0.0827	3	5	positive regulation of cyclic-nucleotide phosphodiesterase activity
GO:0048869	0	2	63	92	3935	cellular developmental process
GO:0005513	0.00E+00	27	0.215	4	13	detection of calcium ion
GO:0009890	0.00E+00	2	25	46	1530	negative regulation of biosynthetic process
GO:0034622	1.00E-04	2	18	35	1064	cellular protein-containing complex assembly
GO:0040037	1.00E-04	22	0.2481	4	15	negative regulation of fibroblast growth factor receptor signaling pathway
GO:0070848	1.00E-04	2	11	26	694	response to growth factor
GO:0045934	1.00E-04	2	23	42	1387	negative regulation of nucleobase-containing compound metabolic process
GO:1903706	1.00E-04	3	5	15	309	regulation of hemopoiesis
GO:0016525	1.00E-04	5	2	10	144	negative regulation of angiogenesis
GO:0043588	1.00E-04	3	7	18	405	skin development
GO:0002027	2.00E-04	6	2	8	95	regulation of heart rate
GO:0060249	2.00E-04	3	7	18	418	anatomical structure homeostasis
GO:1905563	2.00E-04	16	0.3143	4	19	negative regulation of vascular endothelial cell proliferation
GO:0060316	2.00E-04	36	0.1323	3	8	positive regulation of ryanodine-sensitive calcium-release channel activity
GO:1902949	2.00E-04	36	0.1323	3	8	positive regulation of tau-protein kinase activity
GO:0086065	3.00E-04	8	0.8932	6	54	cell communication involved in cardiac conduction
GO:0006583	3.00E-04	Inf	0.0331	2	2	melanin biosynthetic process from tyrosine
GO:0015787	3.00E-04	Inf	0.0331	2	2	UDP-glucuronic acid transmembrane transport
GO:0015789	3.00E-04	Inf	0.0331	2	2	UDP-N-acetylgalactosamine transmembrane transport
GO:0010801	3.00E-04	15	0.3308	4	20	negative regulation of peptidyl-threonine phosphorylation
GO:0002251	3.00E-04	10	0.5954	5	36	organ or tissue specific immune response
GO:0048588	3.00E-04	4	4	12	219	developmental cell growth
GO:1901343	3.00E-04	4	3	10	158	negative regulation of vasculature development
GO:0060429	3.00E-04	2	21	37	1240	epithelium development
GO:1901844	3.00E-04	30	0.1489	3	9	regulation of cell communication by electrical coupling involved in cardiac conduction
GO:0060560	4.00E-04	3	4	12	223	developmental growth involved in morphogenesis
GO:0006941	4.00E-04	4	3	10	165	striated muscle contraction
GO:0003013	5.00E-04	2	9	20	523	circulatory system process
GO:0060047	5.00E-04	3	4	13	264	heart contraction
GO:0019731	5.00E-04	9	0.6616	5	40	antibacterial humoral response
GO:0060315	5.00E-04	26	0.1654	3	10	negative regulation of ryanodine-sensitive calcium-release channel activity
GO:0090287	5.00E-04	3	4	13	267	regulation of cellular response to growth factor stimulus
GO:0009913	7.00E-04	3	6	15	347	epidermal cell differentiation
GO:0055006	8.00E-04	5	2	7	91	cardiac cell development
GO:1903522	8.00E-04	3	5	13	280	regulation of blood circulation
GO:0035456	8.00E-04	11	0.43	4	26	response to interferon-beta
GO:0007494	9.00E-04	20	0.1985	3	12	midgut development
GO:0010800	0.001	10	0.4466	4	27	positive regulation of peptidyl-threonine phosphorylation
GO:0061844	0.001	6	1	6	69	antimicrobial humoral immune response mediated by antimicrobial peptide
GO:0002063	0.0011	7	0.7939	5	48	chondrocyte development

GO:0072359	0.0011	2	18	32	1090	circulatory system development
GO:0003231	0.0014	4	2	8	129	cardiac ventricle development
GO:0043270	0.0014	3	4	12	261	positive regulation of ion transport
GO:0021888	0.0016	60	0.0662	2	4	hypothalamus gonadotrophin-releasing hormone neuron development
GO:0060364	0.0016	60	0.0662	2	4	frontal suture morphogenesis
GO:1990569	0.0016	60	0.0662	2	4	UDP-N-acetylglucosamine transmembrane transport
GO:0055117	0.0016	5	1	6	76	regulation of cardiac muscle contraction
GO:0110110	0.0017	5	1	6	77	positive regulation of animal organ morphogenesis
GO:1901841	0.0018	15	0.2481	3	15	regulation of high voltage-gated calcium channel activity
GO:0048519	0.0018	2	57	77	3640	negative regulation of biological process
GO:0007166	0.002	2	26	41	1658	cell surface receptor signaling pathway
GO:0042633	0.002	4	2	7	107	hair cycle
GO:0031128	0.0021	8	0.5458	4	33	developmental induction
GO:0007167	0.0021	2	16	29	993	enzyme linked receptor protein signaling pathway
GO:0051284	0.0021	14	0.2646	3	16	positive regulation of sequestering of calcium ion
GO:1903587	0.0023	8	0.5624	4	34	regulation of blood vessel endothelial cell proliferation involved in sprouting angiogenesis
GO:0001942	0.0025	5	1	6	83	hair follicle development
GO:2000026	0.0026	2	24	38	1573	regulation of multicellular organismal development
GO:0006599	0.0026	40	0.0827	2	5	phosphagen metabolic process
GO:0046314	0.0026	40	0.0827	2	5	phosphocreatine biosynthetic process
GO:0071504	0.0026	40	0.0827	2	5	cellular response to heparin
GO:1900020	0.0026	40	0.0827	2	5	positive regulation of protein kinase C activity
GO:0006281	0.0028	2	9	18	523	DNA repair
GO:0022404	0.0028	5	1	6	85	molting cycle process
GO:0006334	0.0029	5	1	6	93	nucleosome assembly
GO:0032835	0.0029	6	0.9758	5	59	glomerulus development
GO:0010523	0.003	12	0.2977	3	18	negative regulation of calcium ion transport into cytosol
GO:0031424	0.003	3	4	10	214	keratinization
GO:1902742	0.0031	7	0.612	4	37	apoptotic process involved in development
GO:0043583	0.0034	3	4	10	217	ear development
GO:0051172	0.0037	2	14-Jan	25	918	negative regulation of nitrogen compound metabolic process
GO:0006029	0.0038	4	1	6	90	proteoglycan metabolic process
GO:2000826	0.0038	7	0.6451	4	39	regulation of heart morphogenesis
GO:0021872	0.0038	5	1	5	63	forebrain generation of neurons
GO:0003278	0.0039	30	0.0992	2	6	apoptotic process involved in heart morphogenesis
GO:0060729	0.0039	30	0.0992	2	6	intestinal epithelial structure maintenance
GO:0007219	0.004	3	3	9	187	Notch signaling pathway
GO:0034765	0.0041	2	8	16	458	regulation of ion transmembrane transport
GO:0032516	0.0041	11	0.3308	3	20	positive regulation of phosphoprotein phosphatase activity
GO:1901184	0.0042	4	2	6	92	regulation of ERBB signaling pathway
GO:0048568	0.0042	2	7	15	418	embryonic organ development
GO:0045165	0.0044	3	4	11	262	cell fate commitment
GO:0060485	0.0045	3	Jan-00	11	263	mesenchyme development
GO:1900087	0.0046	7	0.6781	4	41	positive regulation of G1/S transition of mitotic cell cycle

GO:0003002	0.0047	2	6	13	342	regionalization
GO:0070887	0.0048	1	51	69	3104	cellular response to chemical stimulus
GO:0010881	0.0048	10	0.3473	3	21	regulation of cardiac muscle contraction by regulation of the release of sequestered calcium ion
GO:0044342	0.0048	10	0.3473	3	21	type B pancreatic cell proliferation
GO:2000738	0.0048	10	0.3473	3	21	positive regulation of stem cell differentiation
GO:0034764	0.0049	3	3	9	193	positive regulation of transmembrane transport
GO:0071773	0.005	3	3	8	159	cellular response to BMP stimulus
GO:0048585	0.0051	2	26	40	1595	negative regulation of response to stimulus
GO:0035637	0.0053	3	3	9	195	multicellular organismal signaling
GO:0060322	0.0053	2	12	22	733	head development
GO:0051966	0.0053	5	1	5	68	regulation of synaptic transmission, glutamatergic
GO:1904427	0.0053	5	1	5	68	positive regulation of calcium ion transmembrane transport
GO:0006600	0.0054	24	0.1158	2	7	creatine metabolic process
GO:0032808	0.0054	24	0.1158	2	7	lacrimal gland development
GO:0050725	0.0054	24	0.1158	2	7	positive regulation of interleukin-1 beta biosynthetic process
GO:0060449	0.0054	24	0.1158	2	7	bud elongation involved in lung branching
GO:2000020	0.0054	24	0.1158	2	7	positive regulation of male gonad development
GO:0003206	0.0054	4	2	7	128	cardiac chamber morphogenesis
GO:0042472	0.0055	4	2	6	97	inner ear morphogenesis
GO:0051963	0.0055	4	2	6	97	regulation of synapse assembly
GO:0051962	0.0056	2	9	17	516	positive regulation of nervous system development
GO:0032414	0.0057	4	2	6	98	positive regulation of ion transmembrane transporter activity
GO:0051147	0.0062	3	3	8	165	regulation of muscle cell differentiation
GO:2001257	0.0062	3	3	8	165	regulation of cation channel activity
GO:0050878	0.0064	2	8	16	480	regulation of body fluid levels
GO:0035304	0.0064	3	2	7	132	regulation of protein dephosphorylation
GO:0031018	0.0064	6	0.7443	4	45	endocrine pancreas development
GO:0050919	0.0064	6	0.7443	4	45	negative chemotaxis
GO:0001935	0.0067	3	3	8	167	endothelial cell proliferation
GO:0009612	0.0068	3	3	9	203	response to mechanical stimulus
GO:0030857	0.0069	6	0.7608	4	46	negative regulation of epithelial cell differentiation
GO:0007267	0.007	2	26	39	1575	cell-cell signaling
GO:0021979	0.0071	20	0.1323	2	8	hypothalamus cell differentiation
GO:0042756	0.0071	20	0.1323	2	8	drinking behavior
GO:0061476	0.0071	20	0.1323	2	8	response to anticoagulant
GO:0072033	0.0071	20	0.1323	2	8	renal vesicle formation
GO:0003012	0.0073	2	7	15	444	muscle system process
GO:0007610	0.0073	2	9	18	574	behavior
GO:0042059	0.0075	6	0.7774	4	47	negative regulation of epidermal growth factor receptor signaling pathway
GO:0007596	0.0075	2	5	12	322	blood coagulation
GO:0051924	0.0078	3	4	10	245	regulation of calcium ion transport
GO:0031954	0.0079	8	0.4135	3	25	positive regulation of protein autophosphorylation
GO:1903792	0.0079	8	0.4135	3	25	negative regulation of anion transport
GO:0071900	0.008	2	8	16	492	regulation of protein serine/threonine kinase activity

GO:0010648	0.008	2	22	34	1338	negative regulation of cell communication
GO:0055010	0.0081	5	0.7939	4	48	ventricular cardiac muscle tissue morphogenesis
GO:0023057	0.0084	2	22	34	1342	negative regulation of signaling
GO:0007612	0.0087	3	2	7	140	learning
GO:0007268	0.0089	2	11	20	676	chemical synaptic transmission
GO:0051384	0.0091	3	2	7	141	response to glucocorticoid
GO:0060923	0.0091	17	0.1489	2	9	cardiac muscle cell fate commitment
GO:0090647	0.0091	17	0.1489	2	9	modulation of age-related behavioral decline
GO:1990504	0.0091	17	0.1489	2	9	dense core granule exocytosis
GO:2000179	0.0093	5	0.827	4	50	positive regulation of neural precursor cell proliferation
GO:0061138	0.0094	3	3	8	177	morphogenesis of a branching epithelium
GO:0045686	0.0098	8	0.4466	3	27	negative regulation of glial cell differentiation
GO:0060384	0.0098	8	0.4466	3	27	innervation
GO:0086011	0.0098	8	0.4466	3	27	membrane repolarization during action potential
GO:1904893	0.0098	8	0.4466	3	27	negative regulation of STAT cascade
GO:2001025	0.0098	8	0.4466	3	27	positive regulation of response to drug
GO:0099537	0.0099	2	11	20	683	trans-synaptic signaling
GO:0022607	0.0099	1	48	64	2930	cellular component assembly
GO:0002090	0.01	5	0.8435	4	51	regulation of receptor internalization
GO:0043949	0.01	5	0.8435	4	51	regulation of cAMP-mediated signaling
GO:0032410	0.01	4	1	5	79	negative regulation of transporter activity
GO:0048708	0.01	4	1	5	79	astrocyte differentiation
GO:0061061	0.01	2	11	19	638	muscle structure development
GO:0048738	0.01	3	4	9	216	cardiac muscle tissue development
**(b) EGFR: amplified versus non-amplified: Hypermethylated Pathways**						
**GOMFID**	**Pvalue**	**OddsRatio**	**ExpCount**	**Count**	**Size**	**Term**
GO:0050906	0	7	7	39	434	detection of stimulus involved in sensory perception
GO:0050911	0	7	6	34	336	detection of chemical stimulus involved in sensory perception of smell
GO:0009593	0	6	7	37	418	detection of chemical stimulus
GO:0007606	0	6	7	37	431	sensory perception of chemical stimulus
GO:0007186	0	3	21	58	1214	G protein-coupled receptor signaling pathway
GO:0050877	0	3	22	54	1299	nervous system process
GO:0007154	0	2	103	145	6110	cell communication
GO:0023052	0	2	103	144	6084	signaling
GO:0042531	0	9	1	9	67	positive regulation of tyrosine phosphorylation of STAT protein
GO:1904894	0	8	1	10	87	positive regulation of STAT cascade
GO:0032501	0	2	114	151	7001	multicellular organismal process
GO:0070098	0	7	1	9	82	chemokine-mediated signaling pathway
GO:0051716	0	2	119	153	7024	cellular response to stimulus
GO:1990868	0	7	2	9	91	response to chemokine
GO:0046425	0	6	2	9	96	regulation of JAK-STAT cascade
GO:0006959	0	4	4	14	233	humoral immune response
GO:0007166	1.00E-04	2	47	73	2786	cell surface receptor signaling pathway
GO:0031424	1.00E-04	4	4	13	214	keratinization

GO:0009913	1.00E-04	3	6	17	347	epidermal cell differentiation
GO:0045683	2.00E-04	17	0.3042	4	18	negative regulation of epidermis development
GO:0002690	5.00E-04	5	1	7	83	positive regulation of leukocyte chemotaxis
GO:1903670	5.00E-04	5	2	8	109	regulation of sprouting angiogenesis
GO:0043588	6.00E-04	3	7	17	405	skin development
GO:0002252	6.00E-04	2	19	34	1114	immune effector process
GO:0045662	6.00E-04	12	0.4056	4	24	negative regulation of myoblast differentiation
GO:0048584	7.00E-04	2	38	58	2266	positive regulation of response to stimulus
GO:0048483	8.00E-04	8	0.7436	5	44	autonomic nervous system development
GO:0006953	0.001	7	0.7774	5	46	acute-phase response
GO:0030857	0.001	7	0.7774	5	46	negative regulation of epithelial cell differentiation
GO:0002250	0.001	3	7	16	391	adaptive immune response
GO:0002685	0.0011	3	3	10	183	regulation of leukocyte migration
GO:0010273	0.0012	18	0.2197	3	13	detoxification of copper ion
GO:0045766	0.0012	3	3	10	185	positive regulation of angiogenesis
GO:0006954	0.0016	2	12	24	733	inflammatory response
GO:0006968	0.0017	6	0.8619	5	51	cellular defense response
GO:0002361	0.0017	59	0.0676	2	4	CD4-positive, CD25-positive, alpha-beta regulatory T cell differentiation
GO:0003332	0.0017	59	0.0676	2	4	negative regulation of extracellular matrix constituent secretion
GO:0044278	0.0017	59	0.0676	2	4	cell wall disruption in other organism
GO:0045608	0.0017	59	0.0676	2	4	negative regulation of inner ear auditory receptor cell differentiation
GO:0045632	0.0017	59	0.0676	2	4	negative regulation of mechanoreceptor differentiation
GO:0072086	0.0017	59	0.0676	2	4	specification of loop of Henle identity
GO:0045087	0.0018	2	15	27	871	innate immune response
GO:0045747	0.0018	6	0.8788	5	52	positive regulation of Notch signaling pathway
GO:0050679	0.0018	3	3	10	195	positive regulation of epithelial cell proliferation
GO:1901342	0.0018	3	6	15	374	regulation of vasculature development
GO:0097501	0.0019	15	0.2535	3	15	stress response to metal ion
GO:0002684	0.0021	2	17	30	1015	positive regulation of immune system process
GO:0007379	0.0023	14	0.2704	3	16	segment specification
GO:0045321	0.0024	2	20	33	1162	leukocyte activation
GO:0048006	0.0028	39	0.0845	2	5	antigen processing and presentation, endogenous lipid antigen via MHC class Ib
GO:0072047	0.0028	39	0.0845	2	5	proximal/distal pattern formation involved in nephron development
GO:0006882	0.0028	8	0.5915	4	35	cellular zinc ion homeostasis
GO:1903589	0.0032	12	0.3042	3	18	positive regulation of blood vessel endothelial cell proliferation involved in sprouting angiogenesis
GO:0001934	0.0035	2	16	28	961	positive regulation of protein phosphorylation
GO:0042110	0.0037	2	7	16	443	T cell activation
GO:1903546	0.0041	29	0.1014	2	6	protein localization to photoreceptor outer segment
GO:2000503	0.0041	29	0.1014	2	6	positive regulation of natural killer cell chemotaxis
GO:0048713	0.0041	7	0.6591	4	39	regulation of oligodendrocyte differentiation
GO:0032943	0.0043	3	4	11	256	mononuclear cell proliferation
GO:0097696	0.0045	5	1	5	66	STAT cascade
GO:1904888	0.0048	5	1	5	65	cranial skeletal system development
GO:0048514	0.005	2	11	20	627	blood vessel morphogenesis

GO:0071294	0.0051	10	0.3549	3	21	cellular response to zinc ion
GO:0035295	0.0056	2	18	29	1040	tube development
GO:0021546	0.0057	23	0.1183	2	7	rhombomere development
GO:0048007	0.0057	23	0.1183	2	7	antigen processing and presentation, exogenous lipid antigen via MHC class Ib
GO:0030593	0.0058	4	2	6	96	neutrophil chemotaxis
GO:1904427	0.0058	5	1	5	68	positive regulation of calcium ion transmembrane transport
GO:0061844	0.0062	5	1	5	69	antimicrobial humoral immune response mediated by antimicrobial peptide
GO:0097530	0.0063	3	Jan-00	7	129	granulocyte migration
GO:0070102	0.0066	9	0.3887	3	23	interleukin-6-mediated signaling pathway
GO:0090103	0.0066	9	0.3887	3	23	cochlea morphogenesis
GO:0050670	0.0069	3	3	9	199	regulation of lymphocyte proliferation
GO:0050706	0.0069	6	0.7605	4	45	regulation of interleukin-1 beta secretion
GO:0018108	0.0069	2	6	13	351	peptidyl-tyrosine phosphorylation
GO:0035195	0.0072	2	7	15	434	gene silencing by miRNA
GO:0046426	0.0074	8	0.4056	3	24	negative regulation of JAK-STAT cascade
GO:0060396	0.0074	8	0.4056	3	24	growth hormone receptor signaling pathway
GO:0001781	0.0075	20	0.1352	2	8	neutrophil apoptotic process
GO:0002315	0.0075	20	0.1352	2	8	marginal zone B cell differentiation
GO:0045617	0.0075	20	0.1352	2	8	negative regulation of keratinocyte differentiation
GO:2000980	0.0075	20	0.1352	2	8	regulation of inner ear receptor cell differentiation
GO:0043331	0.0075	6	0.7774	4	46	response to dsRNA
GO:0072358	0.0075	2	13	22	740	cardiovascular system development
GO:0002682	0.0078	2	7	14	418	regulation of immune system process
GO:0071715	0.0087	5	0.8112	4	48	icosanoid transport
GO:0045937	0.0087	2	18	29	1076	positive regulation of phosphate metabolic process
GO:0016441	0.0089	2	8	15	445	posttranscriptional gene silencing
GO:0009954	0.0091	8	0.4364	3	26	proximal/distal pattern formation
GO:0043501	0.0093	8	0.4394	3	26	skeletal muscle adaptation
GO:0051482	0.0093	8	0.4394	3	26	positive regulation of cytosolic calcium ion concentration involved in phospholipase C-activating G protein-coupled signaling pathway
GO:1900740	0.0093	8	0.4394	3	26	positive regulation of protein insertion into mitochondrial membrane involved in apoptotic signaling pathway
GO:1901623	0.0093	8	0.4394	3	26	regulation of lymphocyte chemotaxis
GO:0072507	0.0094	2	8	16	490	divalent inorganic cation homeostasis
GO:0010533	0.0095	17	0.1521	2	9	regulation of activation of Janus kinase activity
GO:0034382	0.0095	17	0.1521	2	9	chylomicron remnant clearance
GO:0072048	0.0095	17	0.1521	2	9	renal system pattern specification
GO:0098953	0.0095	17	0.1521	2	9	receptor diffusion trapping
GO:0098970	0.0095	17	0.1521	2	9	postsynaptic neurotransmitter receptor diffusion trapping
GO:0021545	0.01	5	0.845	4	50	cranial nerve development
GO:0021879	0.01	5	0.845	4	50	forebrain neuron differentiation
