# Supplementary figures and images for: Dissecting the Methylomes of EGFR-Amplified Glioblastoma Reveals Altered DNA Replication and Packaging, and Chromatin and Gene Silencing Pathways
Source: Cancers (Basel). 2023 Jul 7;15(13):3525. doi: 10.3390/cancers15133525 (PMC10341082; doi:10.3390/cancers15133525)

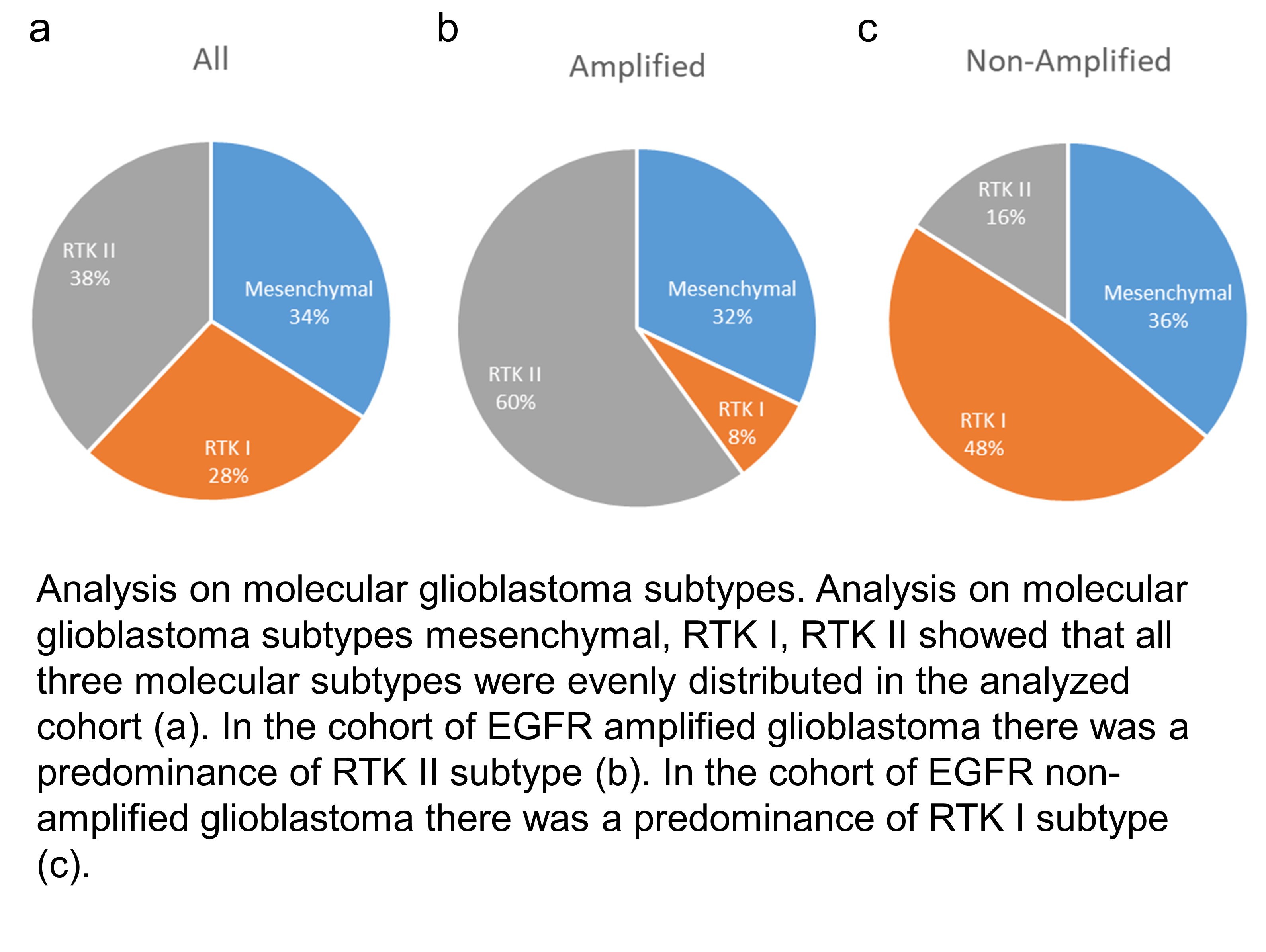

Supplement: Supplementary file 1 [file cancers-15-03525-s001.zip › Figure_S1.TIF]
